# From promise to practice: Navigating the challenges of AI integration in clinical neurophysiology

**DOI:** 10.1016/j.cnp.2026.07.008

**Published:** 2026-07-10

**Authors:** Nortina Shahrizaila, Benjamin H. Brinkmann

**Affiliations:** a Neurology Unit, Department of Medicine, Faculty of Medicine, Universiti Malaya, Kuala Lumpur, Malaysia; b Departments of Biomedical Engineering and Neurology, Mayo Clinic, Rochester, MN, USA

**Keywords:** Artificial intelligence, Clinical neurophysiology, EEG, EMG, Ethical, Risks

## Abstract

The recent position statement by the International Federation of Clinical Neurophysiology (IFCN) provides a critical roadmap for the implementation of artificial intelligence (AI) in clinical neurophysiology. AI has immense potential to augment existing practice, but careful navigation is required to overcome challenges to efficient and ethical clinical deployment. The challenges – including fragmented workflow, opaque governance, ethical liabilities and automation bias – are critically reviewed and strategies are proposed to ensure the seamless transition to widespread implementation within real-world clinical neurophysiology practice.

## Highlights

• Challenges to real-world AI integration include fragmented workflow and governance.
• Strong leadership, good resource, staff and patient buy-ins are essential for success.
• Equitable deployment must mitigate gaps and bias through global collaboration.

## 1 Introduction

Artificial intelligence (AI) is rapidly transitioning from a theoretical research interest to a tangible presence in the clinical neurophysiology laboratory. Currently, the trajectory of innovation is steepest in electroencephalography (EEG), where commercial AI-driven tools are already being deployed to automate seizure detection, spike sorting, and sleep staging.(Koren et al., 2025; Tveit et al., 2023) Conversely, AI solutions for electromyography (EMG) and nerve conduction studies (NCS) are gradually making breakthroughs but have yet to reach the level of diagnostic confidence required for widespread clinical deployment. (Gorenshtein et al., 2025a) Table 1 includes a list of AI tools for clinical neurophysiology currently with or nearing regulatory approval for clinical use.

**Table 1:** Examples of widely available clinical AI tools serving major domains of clinical neurophysiology. Several systems are United States Food and Drug Administration (FDA) cleared and through the European Union's Conformité Européenne (CE) marked in conjunction with specialised acquisition hardware.

Modality and setting	Tool	Capabilities	Validation evidence	Regulatory status
Scalp EEG — routine & continuous ICU (cEEG)	Persyst 15 (seizure & spike detection, qEEG trends, artifact reduction)	Event-level automated seizure detection, spike marking, quantitative EEG trending, real-time artifact reduction	Seizure detector reported non-inferior to expert reviewers; large critical-care comparison (∼7900 records). (Ganguly et al., 2022)	FDA Cleared SaMD, CE marked
Scalp EEG — routine, long-term monitoring (LTM) & ambulatory	Natus autoSCORE 2.0 (commercial implementation of SCORE-AI; developed by Holberg EEG)	Study-level EEG interpretation: normal vs abnormal, then classifies abnormalities as focal- or generalised-epileptiform and focal- or diffuse-nonepileptiform	CNN trained on >30,000 expertly labeled EEGs (17 annotators); validated on 3 independent test sets; accuracy on par with experts. (Tveit et al., 2023)	FDA Cleared SaMD, CE marked
Scalp EEG — routine & continuous ICU (cEEG)	encevis (AIT Austrian Institute of Technology; integrated in Zeto and other platforms)	Spike/IED detection (DeepSpike), seizure detection, electrographic status epilepticus, seizure burden, qEEG and aEEG trending	Independent retrospective comparative validation of encevis 2.0 vs Persyst 14 (Koren et al., 2025). DeepSpike trained on ∼447,000 labeled epochs; (Fürbass et al., 2020)	FDA Cleared SaMD, CE marked
Point-of-care rapid-response EEG (reduced montage)	Ceribell Clarity / ClarityPro	Seizure detection, ESE diagnosis, seizure burden; delirium monitoring	CLARITY retrospective study (353 pts., 6 sites) (Kamousi et al., 2021) Neonatal set >700 recordings.(Gupta et al., 2025)	FDA Cleared — Clarity, neonatal (2025); ClarityPro for ESE cleared 2023. First tool cleared for ESE.
Wireless / wearable ambulatory (home) scalp EEG	Epitel REMI + Vigilenz AI	Extended home EEG recording with AI marking of candidate electrographic seizures	Vendor-reported ML validation; limited independent peer-reviewed data presently.	FDA Cleared (2024)
Wrist wearable (accelerometry, EDA, PPG, temp)	Empatica Embrace2 / EpiMonitor	Detection and caregiver alerting for generalized tonic-clonic seizures	Prospective EMU trials (135–141 pts) vs expert video-EEG published (Onorati et al., 2021)	FDA Cleared Embrace2 (adults 2018; children ≥ 6, 2019); EpiMonitor/EmbracePlus (2024). CE marked
Polysomnography (PSG) & home sleep apnea testing (HSAT)	EnsoData EnsoSleep	Automated sleep staging plus respiratory-event and arousal scoring	Accuracy comparable to registered-technologist interobserver agreement; AASM autoscoring certification pilot ongoing.	FDA Cleared SaMD (2017); (2021, ages 13+, pediatric + expanded events).
Home sleep apnea testing — respiratory inductance plethysmography	Nox DeepResp	Sleep staging & arousal scoring from RIP-belt signals to improve AHI accuracy without EEG	Large validation dataset; improved AHI classification vs standard HSAT.	FDA Cleared, AI-enabled device (2025–26).
EMG / nerve conduction studies (electrodiagnostics)	INSPIRE multi-agent LLM; (Gorenshtein et al.)	Automated interpretation and structured reporting of NCS + needle EMG, integrating clinical history and findings	200 patients randomised to physician-only and AI+physician interpretation, no difference found	Not cleared for clinical use No regulatory pathway yet; no commercial product.
				

cEEG, continuous EEG; FDA, Food and Drug Administration; CE, Conformit´e Europ´eenne; LTM, long-term monitoring; CNN, convolutional neural network; ML, machine learning; LLM, large language model; ED, emergency department; ICU, intensive care unit; EMU, epilepsy monitoring unit; IED, interictal epileptiform discharge; EDA, electrodermal activity; PPG, photoplethysmo graphy; RIP, respiratory inductance plethysmography; PSG, polysomnography; HSAT, home sleep apnea test; ESE, electrographic status epilepticus; GTCS, generalized tonic -clonic seizure; AHI, apnea–hypopnea index; SaMD, software as a medical device; qEEG/aEEG, quantitative / amplitude -integrated EEG.

Regardless of the modality, the rapid commercialisation of these tools has outpaced standardised clinical workflows. Recognising this gap, the recent International Federation of Clinical Neurophysiology (IFCN) position statement explicitly emphasised the need for the responsible integration of AI. (Husain et al., 2026) The position statement highlights (i) augmentation, not replacement, of neurophysiologists' expertise and clinical judgment; (ii) the need for diverse data and transparent, ethical training; and (iii) prioritisation of patient safety, clinical integrity, and strong scientific standards. To achieve this mandate, it is imperative that the clinical community critically evaluates not just the algorithmic accuracy of these tools, but their real-world impact on the laboratory environment.

In this article, we review the practical, technical, and ethical challenges of AI integration across routine neurophysiology modalities. Specifically, we address the risks of workflow fragmentation, diagnostic liability and automation bias, providing actionable strategies for the safe implementation of AI in daily practice.

## 2 Challenges in integrating AI processes

From a practical perspective, integration of AI into neurophysiology clinical practice brings with it several challenges. The first, and perhaps most daunting challenge, involves the integration of AI software into a fragmented technology ecosystem. Hospitals often rely on systems from different vendors to record neurophysiology, and data storage systems and formats are proprietary and mutually incompatible. Clinicians often prefer to have analytical tools integrated with recording and review systems for ease of use, and it is challenging for AI software companies to integrate with all existing recording systems. This difficulty increases the cost of software, the technical support requirements for hospitals, may add burden to clinicians overseeing clinical studies and makes effective oversight of AI systems more difficult.

Further challenges exist in the data infrastructure and adaptation of workflows to support AI deployment at scale. It is relatively simple to apply most neurophysiology AI tools to small datasets for clearly defined analyses. However, true integration and full-scale deployment require AI inference to be computed on multiple large datasets before human expert review. AI tools need to run autonomously on datasets on large-scale Storage Area Network (SAN) or Cloud storage systems, and AI tools need to be configured to run on a central server with little or no user input. Persistent oversight, by IT staff or perhaps in future by autonomous AI agents, is needed in order to restart failed processes or deal with intermittent problems to ensure study processing proceeds unhindered in a timely manner. In a hospital environment, process uniformity is essential to ensure uniformly high quality for all studies; therefore, optimising input parameters for AI algorithms individually for each clinical case is not feasible. This means that AI algorithms must be robust to common artifacts and differences between subjects and recording systems, and the accuracy of AI algorithm output can degrade if the quality and consistency of the neurophysiology data acquired decreases.

Integration of AI tools across different modalities and into the electronic health records (EHR) is even more challenging, and not yet in scope for most vendors. Emerging AI tools are modality specific, and shared toolkits between EEG, magnetoencephalography (MEG), transcranial magnetic stimulation (TMS), EMG, polysomnography (PSG) and other neurophysiology modalities are hindered by the lack of unified data format and standards. (Halford et al., 2021) Clear, concise, and consistent computer-generated reports describing algorithm output are an important component of the clinical workflow, and having a unified framework for AI algorithm reporting will be important to support interpretability and clarity. Such reports should call out artifacts and any data issues present and must provide a measure of the confidence of individual AI outputs. Such confidence scores may be derived primarily from likelihood scores generated by algorithms, but also need to account for data quality (noise and artifacts should decrease confidence) and other alternative classifications of neurophysiological features (for example, interictal spike discharges that look similar to wicket waves or other benign variants).

Clinician trust of AI tools is essential to ensure widespread adoption. While conventional analytical tools can be deconstructed to provide an intuitive understanding of the results, most AI algorithms do not provide similar clues to their internal workings. This lack of intuitive feedback impedes trust by clinicians. While some methods exist to extract insights from classifications by deep learning algorithms, (Lundberg and Lee, 2017; Zeiler and Fergus, 2014) these methods can be challenging in practice and are not practical to use with commercially available neurophysiology AI tools. Therefore, clinicians using AI tools in clinical neurophysiology are left to build intuition and trust through experience and repeated use. Well curated datasets containing clinically relevant waveforms may help improve clinician trust and adoption of AI tools, and independent medical organisations could play an important role in developing and maintaining shared validation datasets.

Well-designed and developed models will have been trained using broad representation of data acquired on systems from different vendors, under the full range of clinical conditions, and including a full range of noise and artifact sources. Such models should be relatively free from overfitting and will generalise well in new situations. However, subtle differences in training data sets compared to data analysed in a hospital deployment have the potential to cause errors and biases in AI output and rigorous testing is necessary during initial deployment and on an ongoing basis to ensure that algorithms function accurately and reliably.

## 3 Challenges in data quality and robustness

The fundamental nature of data generation in clinical neurophysiology varies drastically by modality, dictating the unique challenges AI faces in each space. EEG produces massive, continuous datasets where following the skilled manual setup and initiation by a technologist, the recording process itself runs automatically; the primary limitation here lies in the human interpretation of the resulting waveforms. In contrast, NCS and EMG are dynamic and operator-dependent techniques. In these modalities, the quality of the data generated relies entirely on the clinician's real-time technical skills, anatomical knowledge, and clinical interpretation.

Applying machine learning to the high-volume data of an EEG can significantly improve workflow efficiency, identifying epileptiform vs. non-epileptiform discharges so the clinician can focus on clinical correlations. More recently, advanced applications are shifting toward full interpretive support, providing automated clinical correlations and classification text for straightforward, routine cases.(Tveit et al., 2023) To a lesser degree, machine learning could also be applied to NCS and EMG waveforms to differentiate between normal and abnormal ranges, although their dynamic nature makes it challenging to strictly dichotomise these findings.(Gorenshtein et al., 2025b).

However, data quality is compromised by institutional silos and technical interoperability gaps. For instance, models trained on data from Nihon Kohden amplifiers may fail to translate when applied to data collected on Natus or Cadwell systems due to subtle differences in sampling rates or proprietary filter settings. Even if the hardware is identical, human subjectivity remains a confounding variable. Inter-rater reliability among clinicians is also variable; an AI trained on one clinician's subjective grading of an EEG background or seizure onset may be different to the clinical standards of another clinician.(Boe et al., 2010; Halford et al., 2017; Younes et al., 2016).

Furthermore, the efficacy of any model is entirely dependent on its training and validation datasets, and their similarity to routine clinical recordings—which, in reality, are highly variable and fragmented. This variability cuts across health systems, patient demographics, equipment settings, and clinical expertise. For example, the high-quality EEG data acquired in a well-funded academic institution may look vastly different from the data acquired in a rural district hospital struggling with electrical interference and older equipment. By failing to include these under-represented, “real-world” datasets in training algorithms, AI models risk amplifying health disparities across diverse patient populations.

## 4 Ethical and liability considerations

The integration of AI into the practice of clinical neurophysiology spans a spectrum of AI autonomy, human oversight, and role as follows:

• Predictive AI: Machine learning algorithms predict the presence or absence of specific abnormalities based on historical training datasets.
• Generative AI: Algorithms generate clinical reports by analysing input datasets alongside generated predictions.
• Assistive AI: A “human-in-the-loop” model where AI provides diagnostic support and drafts reports, but clinician oversight and the ability to override remain mandatory.
• Autonomous AI: Independent deployment of AI tools—potential utility in resource-limited settings lacking on-site experts—to determine clinical outcomes directly from data acquisition without human intervention.

Whether adopting assistive or autonomous AI models in clinical neurophysiology practice, there are ethical questions and clinical liabilities to be considered. The deployment of AI introduces a complex web of stakeholders, including healthcare institutions, technology developers, clinicians, and patients. Establishing robust AI governance is paramount to ensure these tools remain safe, clinically valid, and respectful of human rights.

Recognising these challenges, global health authorities, including the World Health Organization (WHO), are establishing critical frameworks for the ethical use of AI and large multi-modal models in healthcare. (*Ethics and Governance of Artificial Intelligence for Health: WHO Guidance,* 2021) In the field of epilepsy, the International League Against Epilepsy Global Advocacy Council and Big Data Commission recently published a roadmap outlining broad steps toward ethical, equitable, and effective AI in patient care.(Josephson et al., 2026) These frameworks are highly relevant given the accelerating approval of AI-based EEG interpretation solutions by the U.S. Food and Drug Administration (FDA) and through the European Union (EU)’s Conformité Européenne (CE) marking. However, the integration of these tools into wider clinical neurophysiology practice raises critical ethical and liability issues:

• Patient Consent and Data Sovereignty: The transition to more advanced AI-assisted diagnostics may alter the traditional patient-physician data contract. Utilising personalised, multimodal datasets (combining neurophysiology with imaging, medical history, and treatment records) amplifies privacy and security risks. Guidelines regarding whether or not patients should be informed that AI tools were used in their care are still ambiguous and lack consensus, and the practical implications of accommodating patients who object to the use of AI tools are complicated, as fully integrated tools may not be possible to extricate from clinical workflows. The question of whether patients should be able to opt out of having their data used to retrain AI algorithms is an active question as well, and currently rules are heterogeneous between different regions and regulatory authorities. This is an evolving area, and further work is needed to build consensus on best practices in this area.

• The Accountability Gap: While current AI applications in clinical neurophysiology primarily serve to augment clinician capabilities, the push toward greater autonomy complicates medical liability. In scenarios where autonomous AI is deployed to bridge expert shortages, the legal responsibility for misdiagnosis becomes opaque. This accountability gap is perpetuated by complex mathematical algorithms that are not easily explained by the end-users, introducing ambiguity as to whether liability for an algorithmic error falls on the clinician, the healthcare institution, or the software developer. Malpractice laws for AI-enabled medical diagnostics are evolving, but have not kept up with the fast-changing state of clinical AI (Mello and Guha, 2024; Wachter et al., 2017). Ultimately physicians remain accountable for medical decisions whether AI is used or not, but the black-box nature of many models complicates legal decisions around whether accepting or contradicting an AI categorisation is reasonable, or whether an error by an AI tool indicates a defect or normal variation.

• Regulatory Hurdles and Algorithmic Bias: Given the high stakes of AI in clinical neurology, one could liken the use of AI diagnostic tools in clinical neurophysiology to that of medical devices and therapeutics. This highlights the need for stringent, standardised validation prior to deployment. To achieve regulatory compliance and clinical safety, it stands to reason that AI-based tools should undergo rigorous clinical trials across diverse global population cohorts. This is of greater importance given the variable healthcare infrastructure and distinct demographic data present in different regional settings. Failing to validate these tools across diverse populations risks perpetuating algorithmic bias and compromising cross-cultural clinical validity.

## 5 Automation complacency and deskilling

Neurologists fundamentally rely on their clinical acumen to localise pathology and guide diagnosis. In this process, neurophysiological tools serve as a vital extension of the clinician's toolkit, utilised primarily to narrow differential diagnoses. Just as interpreting a 12‑lead electrocardiogram is foundational for diagnosing acute myocardial ischemia, the art of evaluating raw EEG, MEG, PSG, and NCS/EMG waveforms is an indispensable component of neurological practice. However, the increasing automation of these processes through AI threatens to erode this essential clinical skillset.

There is a growing precedent where clinicians default to accepting AI-generated reports rather than critically analysing the raw neurophysiological datasets, a phenomenon often termed “automation bias” (Khan, 2025). If left unchecked, this over-reliance could circumvent the hands-on experience needed to build competency and severely impact medical training; junior clinicians and trainees may fail to develop the pattern recognition expertise necessary for evaluating complex cases (i.e., deskilling). A further potential risk of over-reliance on AI by trainees is “mis-skilling”, where trainees accept AI errors as truth and learn these waveforms incorrectly. Training programs in particular should use AI carefully to ensure deskilling and mis-skilling do not occur.

Furthermore, unlike some generative AI models that provide a traceable “chain of thought,” the deep neural networks often employed in clinical neurophysiology function largely as “black boxes”. They process high volumes of complex data, but the mechanisms by which they highlight abnormalities are rarely transparent. This lack of explainability makes it profoundly challenging for clinicians to independently verify or justify the AI's diagnostic rationale. (Abdulnour et al., 2025).

In real-world clinical settings, AI models can struggle to distinguish between genuine physiological findings of interest and non-physiological or physiological artifacts. For example, certain artifacts could be erroneously flagged as epileptiform discharges or seizures on EEG, potentially leading to unnecessary interventions.(Acar et al., 2026) In continuous monitoring settings such as in intensive care, over-reliance on AI can breed complacency, perpetuating a false sense of security and diminishing the vigilance required for real-time observation.

Additionally, complex clinical cases frequently demand a more nuanced, “outside-the-box” creative and critical thinking. Given that AI operates on standardised algorithms and historical data, it may be unable to handle these complexities, ultimately compromising patient care. Regardless of the tools employed, the ultimate legal and ethical liability for patient management remains with the attending clinician, underscoring the absolute necessity of maintaining robust, independent human neurophysiological expertise.

## 6 Commercial monopolies and diagnostic outsourcing

Currently, AI development is concentrated within a few commercial entities, creating supply chain vulnerabilities where service interruptions or service provider restructuring could potentially disrupt daily clinical operations. Over-reliance on AI-assisted tools might lead to an outsourcing of diagnostics that limits institutional clinical autonomy, relegating clinical neurophysiologists to merely supervising “black boxes”. Even in the large consumer-facing AI marketplace this is further compounded by a geographic bias whereby AI tools are available to and trained predominantly on high-income Western cohorts and may fail to generalise across diverse populations in other global cohorts.

Taken together, this trajectory risks exacerbating global health inequities and could result in the erosion of expertise, shifting autonomy from independent clinical neurophysiology labs to outsourced commercial monopolies.

## 7 Current frameworks for quality assurance

Several agencies and organisations have released guidelines for quality assurance in AI deployments. The US FDA's guidance on machine learning based software as a medical device (U.S. Food and Drug Administration, 2025) requires AI software vendors to create Predetermined Change Control Plans (PCCPs) that specify how to monitor deployed AI software and what conditions would cause a model to be updated or rebuilt. The US National Institute of Standards and Technology (NIST) has published a first version of an AI Risk Management Framework which specifies four principles for risk minimisation as follows: (National Institute of Standards and Technology, 2023)

• Govern: ensures that appropriate personnel and standard operating procedures are in place to support AI risk management decisions and practices.
• Map: ensures organisations have thoroughly explored likely vulnerabilities in AI software and the consequences of AI errors in the context of its role in the enterprise.
• Measure: refers to a programme of ongoing, regular testing, evaluation, validation, and verification throughout the AI software life cycle in order to detect errors and drift in model performance.
• Manage: ensures that a response plan is in place to deal with errors that are detected either in routine testing or in application of AI models, and includes the assessment of residual risk after mitigation plans are finalised.

**Fig. 1:**
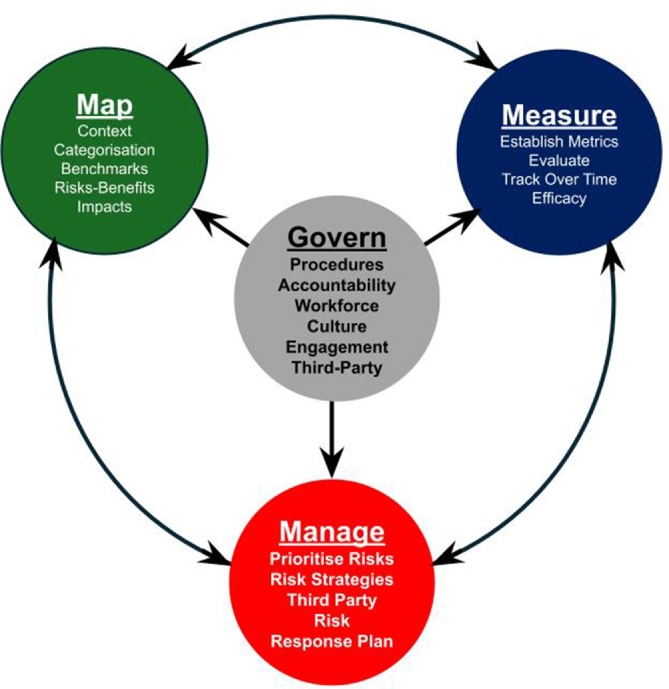
National Institute of Standards and Technology (NIST) AI Risk Management Framework. Risk management with AI requires mutual engagement between vendors and hospitals in a robust framework. The central Governance framework ensures that organisational policies and procedures are in place for managing risk, appropriate teams and individuals are trained, empowered, and accountable to manage risk, workforce equity and accessibility are prioritised in risk management strategies, teams have a culture that supports communication of AI risks, systems support robust engagement by all stakeholders, and risk management includes third party interactions. Mapping activities focus on understanding the context of risks, categorising the AI system's role and limitations, the AI system's capabilities, goals, costs and benefits are benchmarked appropriately, ensuring the risk-benefit equation is understood, and the system's impact on groups, individuals, communities, and society are known. Measurement requires appropriate metrics are in place, systems are evaluated, quantitatively or qualitatively, for trustworthy characteristics, performance metrics are tracked longitudinally over time, and feedback on the efficacy of measurement is collected and reviewed. Management activities ensure that risks are dealt with effectively by prioritising and responding to risks identified in mapping and measurement activities, strategies to minimise risks and maximise benefits from the AI system are planned, implemented, and documented, risks and benefits from third parties are managed, and risk plans include response, recovery, and communication and these plans are documented and monitored.

As a generalised framework the NIST system does not prescribe specific tests to perform nor acceptable levels of deviation from expected outputs, as these parameters depend on the specific application of AI and the consequences of AI errors. (Fig. 1) This publication serves as a comprehensive guide for vendors and healthcare organisations to assess risk and design robust, fault-tolerant AI systems. A companion volume focused on generative AI was released in 2024 (AI, 2024), addressing the particular challenges associated with large language models (LLMs) and generative technologies.

In contrast to the guidelines provided by US agencies, legal requirements have been established by the EU's AI Act, mandating clear risk stratification of applications and rigorous continuous monitoring for all AI systems. (European Parliament, Council of the European Union, 2024) Most medical applications of AI would fall into the high-risk category and would therefore require conformance assessment by a notified body. Typically, such AI applications are subject to extensive post-market monitoring, data governance restrictions, and disclosures to users about the system's limitations. Although the Act was ratified in 2024, full compliance is not required until August 2027, providing some latitude as companies develop frameworks and procedures. Medical AI systems in the EU must comply both with the EU AI Act as well as the existing Medical Device Regulation statutes,(European Parliament, Council of the European Union, 2017) creating a more complicated compliance landscape for AI medical software companies. Several best practice guidelines have been published by industry groups, including the Coalition for Health AI (CHAI)’s Blueprint for Trustworthy AI implementation Guidance, written by a panel of industry experts for hospitals and health care providers struggling to implement appropriate AI governance strategies in the current fast-changing environment. (Coalition for Health AI (CHAI), 2023).

## 8 Practical strategies for AI integration in the “real-world” lab

Despite the potential risks and challenges associated with the integration of AI in clinical neurophysiology, the potential benefits cannot be overlooked. We are entering an era where there is an exponentially increasing demand for the use of AI; for instance, generative AI is now commonplace despite the associated risks of cognitive offloading. Vendors of neurophysiology equipment are increasingly incorporating AI tools into standard equipment, making its adoption inevitable. The aim now is to build a framework that safeguards clinical integrity while navigating the unique local constraints of the real-world lab.

**Fig. 2:**
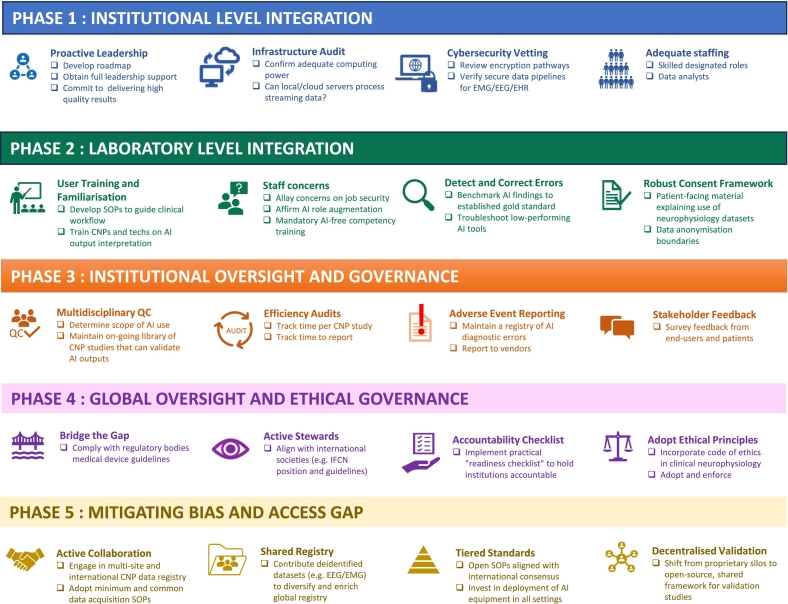
Practical Strategies for AI integration in the “real-world” clinical neurophysiology lab. A framework is proposed encompassing the five key stages that are required to navigate the risks and challenges of AI integration, beginning at the local institutional and laboratory levels and moving through continuous local and international oversight whilst mitigating commercial bias and unequal access. At the institutional level (Phase 1), a proactive strategy with good leadership, ample staffing and robust cybersecurity infrastructure is necessary. Integration in the laboratory (Phase 2) requires users' familiarity with AI systems through regular training whilst also ensuring a system is in place that detects and corrects errors. AI mistrust and job security concerns (i.e. augment not replace) must be addressed through transparent communication and consent. Once integrated, rigorous, continuous monitoring and dynamic governance structure (Phase 3) must be established to ensure an efficient and safe AI-integrated workflow that produces outcomes that meets or exceeds existing clinical standards. High-level frameworks exist on safe AI deployment but professional societies, e.g. IFCN and member societies, can serve as active stewards to advocate and enforce (Phase 4). Finally, to ensure equitable AI practices (Phase 5), collaborative models with shared open-source platforms, a tiered technical core standards of data acquisition and interpretation as well as a decentralised clinical validation framework should be championed.

An overview of the practical strategies for AI integration is depicted in Fig. 2 and the deployment checklist of a hypothetical AI-assisted neurophysiology tool in the “real-world” is shown in Box 1.

**Box 1:**
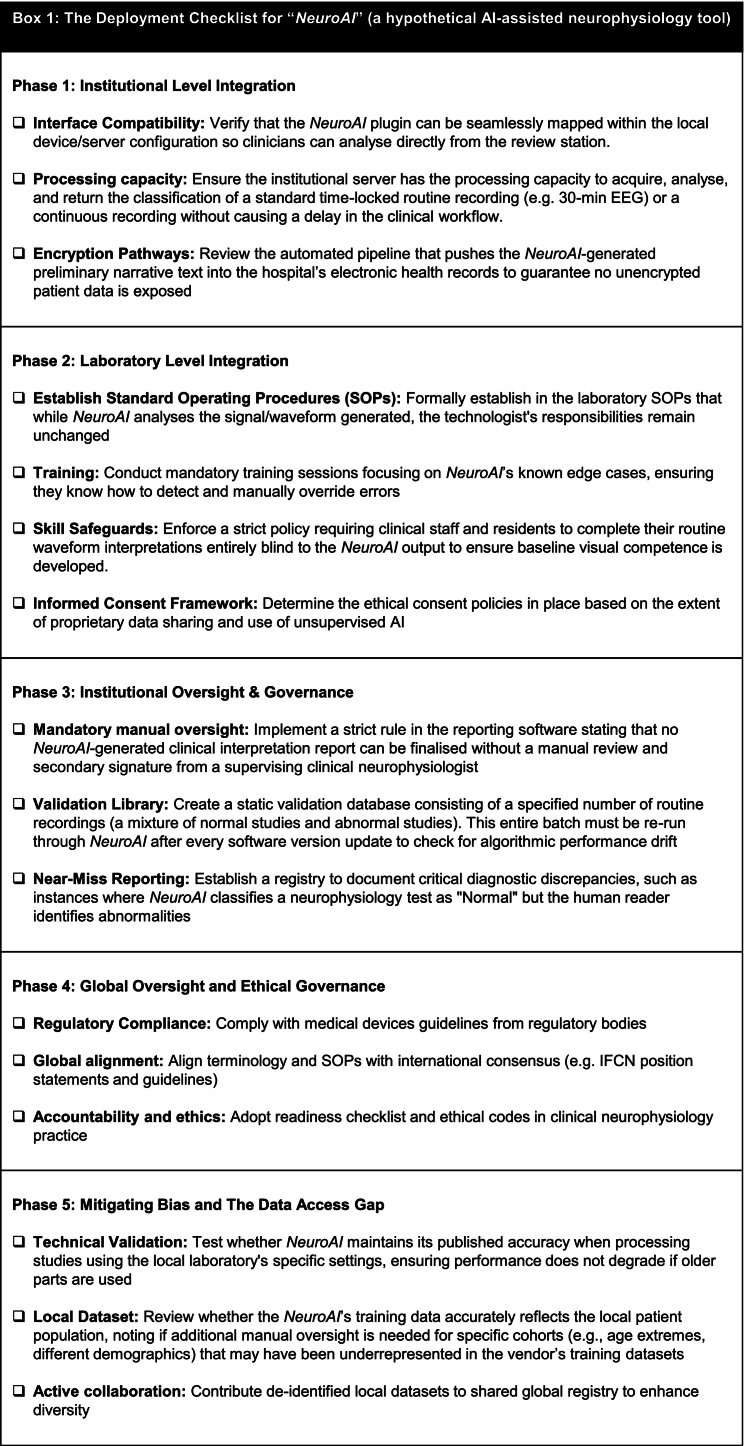
The Deployment Checklist for “NeuroAI” (a hypothetical neurophysiology tool)

**Phase 1: AI integration at the institutional level.**

Successful real-world integration of AI tools at the institutional level requires a proactive comprehensive strategy informed by industry best practices and supported by hospital leadership and resources. AI integration strategies should be tailored to the risk profile of the application and its intended use in the clinic, and AI integration must happen in concert with the hospital's cybersecurity practices and infrastructure. Adequate staffing and support are essential for successful AI integration activities.

Any AI program, no matter how well designed, may become a liability to clinical workflows without well-trained staff, sufficient computing power, and full integration with institutional servers and storage systems. These resources require support from institutional leadership and a commitment to quality.  

**Phase 2: AI integration at the laboratory level.**

Successful AI integration at the individual lab level should include user training and familiarisation. AI deployment plans must consider staff concerns about potential mistrust of AI medical software and job security, and affirm that AI software's purpose is to augment and empower clinical teams, not impede or replace them. All users of AI software must be well informed on detecting and correcting AI errors, and clinical personnel should ensure they have opportunities to practice and reinforce clinical skills and judgment without becoming dependent on AI. To maintain diagnostic transparency, institutions must continuously align their consent frameworks with evolving best practices. Consent should explicitly outline how algorithmic tools leverage patient neurophysiological data for clinical decision-making, while clearly defining the boundaries of data anonymisation when sharing proprietary data with third-party AI developers.

At the lab level, a comprehensive set of standard operating procedures (SOPs) should be developed to guide how lab personnel interact with and document AI tools in clinical workflows. These SOPs should include procedures for assessing the accuracy of AI output and for how to address problems with AI tools performing below the required level of accuracy.  

**Phase 3: Ongoing institutional oversight and governance.**

  Once the initial integration is complete, the focus must shift from implementation to rigorous, ongoing oversight. It will not suffice to have AI-assisted tools in the clinical neurophysiology lab to be left unattended. Much like one would monitor the efficacy of a therapeutic agent when prescribed to patients, the use of AI requires a dynamic governance structure that ensures it remains clinically safe and operationally efficient. In broad terms, this can be achieved at the institutional level through the following:

• Multidisciplinary Quality Control: Moving beyond standard engineering checklists to include collaborative audits between information technology (IT) specialists, AI model experts, and clinicians.
• Workflow and Efficiency Audits: Regular assessments to determine if the AI is truly optimising the pipeline or merely introducing new redundancies.
• Adverse Event Reporting: Establishing a transparent “feedback loop,” modelled after pharmaceutical adverse effect reporting, to identify systematic errors.
• Stakeholder Feedback: Continuous monitoring of patient sentiment and end-user (technician/clinician) feedback to prevent “trust erosion.”

To ensure AI processes continue to deliver high-quality diagnostic support, an ongoing, robust quality control framework is essential. Unlike standard clinical neurophysiology equipment, AI-integrated workflows require more than just a periodic biomedical engineering check. Effective quality control in clinical neurophysiology AI tools requires a collaborative approach that involves IT experts knowledgeable in deep learning models working alongside clinicians to ensure the AI's output consistently meets or exceeds established clinical standards.

In the early stages, this would involve frequent clinical audits where AI-generated results are benchmarked against conventional manual interpretation. Beyond technical accuracy, the institution must audit workflow efficiencies: is the AI reducing the burden on clinicians, or is it creating additional work with data verification? Additionally, a reporting system for AI “near-misses” or errors—similar to adverse event reporting in prescribing pharmaceutical drugs—must be advocated to ensure patient safety remains a priority.

Practical implementation of AI quality control depends heavily on the use and scope of the AI application, but for many clinical neurophysiology applications maintaining an ongoing library of known positive and normal studies which are regularly processed by the AI tool as a validation check forms an essential core element of such a program. Tracking the average time required for physicians and technologists per study over time is important to detect and address workflow inefficiencies that may arise.  

**Phase 4: Global oversight and ethical governance.**

International frameworks like the Declaration of Helsinki and International Council for Harmonisation Good Clinical Practice (ICH-GCP) provide a moral compass for clinical research but their application to AI in clinical neurophysiology remains unclear. While bodies such as the WHO, US FDA and the EU have issued guidance on the use of AI in medicine, the architects of these high-level frameworks are not positioned to oversee their day-to-day implementation in a local neurophysiology laboratory.

To bridge this gap, professional organisations—such as the IFCN and its member societies—should step in as neutral proxies. Rather than acting as passive observers, these societies can serve as active stewards of safe deployment of AI in clinical neurophysiology. The recent IFCN position statement (Husain et al., 2026), for instance, could be utilised as a practical “readiness checklist” to hold institutions accountable, ensure that AI risk management plans are in place, and ensure that ethical principles like justice and beneficence are not just cited, but adopted and enforced.  

**Phase 5: Mitigating Commercial Bias and the Data Access Gap.**

A significant barrier to equitable AI is the resource-intensive nature of its development. The immense financial and technical infrastructure required to train sophisticated models often limits innovation to well-funded institutions and vendors in high-income regions. This creates a commercial bias where tools are designed for the most profitable markets; and a representational bias where the underlying datasets reflect demographics that may not generalise to resource-limited settings.

Current consensus statements often champion the “potential” of AI in limited resource settings without addressing the “access gap.” (Husain et al., 2026; Josephson et al., 2026) Much like specialised neurological treatments that remain out of reach for the communities that need them most, AI tools in clinical neurophysiology risk becoming a luxury of the high-income countries, despite their potential to be highly impactful in improving outcomes in low-resource, underserved regions.

To counter this, the clinical neurophysiology community should look toward the emerging models of collaborative, non-profit consortia in the field of medicine where de-identified clinical datasets and matched biological samples are shared in free-access platforms. (Alzheimer's Disease Neuroimaging Initiative, 2026; Health research data for the world, 2026; Temple University EEG, 2026)

In clinical neurophysiology a similar framework would incorporate the following:

• The Shared Signal Registry: Organisations such as the IFCN and its member societies could facilitate an open-source platform where global laboratories contribute de-identified, standardised data format files. This allows for the training of models on a truly representative global dataset.
• Tiered Technical Standards: By establishing a “minimum core standard” for technical data acquisition and interpretation, the initiative ensures that laboratories in resource-limited settings can participate in and benefit from AI development without requiring cost-prohibitive hardware.
• Decentralised Clinical Validation: Utilising a global network for model validation ensures that machine learning algorithms and pattern recognition tools (such as automated seizure detection) are robust enough to handle the varying recording environments and equipment found in real-world labs worldwide. This shift from proprietary silos to an open-source, shared framework enhances trust and ensures that clinical neurophysiology AI serves the needs of all patients, not just the market. This may be supported by sharing deidentified data in a central repository that labs can access globally for tool validation, and by establishing global user groups to share standard operating procedures and experience.

## 9 Conclusion

With rare exceptions, the integration of AI in clinical neurophysiology currently remains suspended in a state of promise rather than widespread practice. By navigating these operational, ethical, and technical challenges through deliberate, step-wise strategies, the global neurophysiology community can bridge this implementation gap while ensuring that AI deployments are sustainable, beneficial, and ethical. Ultimately, these frameworks ensure that front-line clinicians and technical staff encounter an AI ecosystem that optimises laboratory workflows, enhances diagnostic precision, and reduces clinician burden, resulting in better patient care while actively safeguarding and enriching core clinical expertise.

## Funding

This work was commissioned and did not receive any funding.

## Declaration of competing interest

Nortina Shahrizaila reports no competing interest. Benjamin H. Brinkmann reports the following: Grants from National Institutes of Health, UNEEG Medical, Epilepsy Foundation of America; Royalties from Cadence Neuroscience; honoraria from Eisai Inc. and UCB Inc.; support for attending meetings from Freiburg University; patents for brain impedance, multiscale electrodes, wearable devices; co-chair ILAE Neurotechnology section (uncompensated)
